# Safety and efficacy of rituximab in refractory noninfectious uveitis and scleritis

**DOI:** 10.1186/s12348-025-00522-5

**Published:** 2025-09-26

**Authors:** Faris M. Al Ghulaiga, Ibrahim Alharbi, Abdulrahman F. Albloushi, Abdulsalam M. Dheyab, Marwan A. Abouammoh, Ahmed M. Abu El-Asrar

**Affiliations:** 1 Department of Ophthalmology, College of Medicine, King Saud University, P.O.Box 245, Riyadh, 11411 Saudi Arabia; 2 Arab National Bank Research Chair in Ophthalmology, College of Medicine, King Saud University, Riyadh, Saudi Arabia

**Keywords:** Granulomatous uveitis, Rituximab, Vogt-Koyanagi-Harada disease, Refractory uveitis

## Abstract

**Purpose:**

To investigate the safety and efficacy of rituximab in patients with refractory noninfectious uveitis and scleritis.

**Methods:**

A retrospective review of 24 patients (47 eyes) treated with rituximab for refractory noninfectious uveitis and scleritis in the period between January 2018 and December 2024. The diagnosis of these patients included chronic recurrent uveitis associated with Vogt-Koyanagi-Harada disease (*n* = 16), idiopathic granulomatous uveitis (*n* = 3), multiple sclerosis-associated uveitis (*n* = 2), progressive subretinal fibrosis (*n* = 1) and scleritis (*n* = 2). The primary outcomes were disease quiescence and improvement in best-corrected visual acuity (BCVA). Secondary outcomes included reduction in corticosteroid and immunosuppressive therapy and adverse events.

**Results:**

Rituximab was successful in inducing and maintaining disease quiescence and significantly improved visual acuity in patients presenting with uveitis and scleritis. After starting rituximab therapy, BCVA improved at 1 year from 0.93 ± 0.67 logMAR (Snellen: 20/160) to 0.63 ± 0.83 logMAR (Snellen: 20/80) (*p* < 0.001). Follow-up period ranged from 12 to 80 months (mean 41 ± SD 20.5). At the last follow-up, BCVA continued to improve to 0.46 ± 0.73 logMAR (Snellen: 20/60) (*p* < 0.001). Nine of twenty-four patients (37.5%) required more than 3 rituximab doses for disease control. Rituximab successfully reduced corticosteroid use, allowing 87.5% (21/24) of patients to discontinue steroids completely, with the remaining needing ≤ 7.5 mg/day. Mycophenolate mofetil dosage significantly decreased from a mean of 1840 ± SD 323 mg to 1045 ± SD 688 mg daily (*p* < 0.001), with two patients discontinuing all medications. All patients were flare-free at last follow-up.

**Conclusions:**

Rituximab is an effective and safe treatment for refractory noninfectious uveitis and scleritis. It offers significant visual improvement, disease quiescence which potentially reduces reliance on corticosteroids and immunomodulatory therapy.

## Introduction

Autoimmune uveitis is a significant cause of visual impairment, contributing to approximately 10% of global blindness cases [1]. Granulomatous uveitis, a subtype that can be idiopathic or associated with systemic diseases such as sarcoidosis, Vogt-Koyanagi-Harada (VKH) disease, and multiple sclerosis (MS), accounts for 10–50% of all uveitis cases [2]. If untreated or inadequately managed, granulomatous uveitis can lead to severe complications, including cataract, glaucoma, macular edema, retinal detachment, and optic nerve damage, culminating in substantial vision loss [3].

Conventional treatment using high-dose systemic corticosteroids has been shown to be ineffective in the treatment of initial-onset acute uveitic phase of VKH disease. However, the addition of immunomodulatory therapy, such as mycophenolate mofetil combined with systemic corticosteroids in patients with acute initial-onset disease has been shown to prevent the progression to the chronic phase with “sunset-glow” fundus, reduce late complications, and avoid the development of vitiligo and poliosis. Moreover, patients undergoing this combined therapy were able to discontinue treatment without experiencing a relapse of inflammation [4, 5]. These findings suggest that there is a critical therapeutic window during the early acute uveitic phase when treatment can be most effective, likely because the disease process has not yet fully matured [6–9]. In the case of inadequate treatment in the initial-onset acute VKH disease, or if patients present in the initial-onset delayed phase, patients will convert to chronic recurrent VKH disease which manifests more severe granulomatous inflammation of the anterior segment and may develop vision-threatening complications, namely cataract, glaucoma, subretinal neovascular membranes subretinal fibrosis and “sunset-glow” fundus [10]. Aqueous humor fluorometry showed that both aqueous flare values and cell counts were significantly higher in chronic recurrent disease than those in initial-onset acute disease. In addition, chronic recurrent granulomatous inflammation is more refractory to treatment, needing prolonged and repeated therapeutic interventions [11].

Several studies have highlighted the potential role of B lymphocytes in the pathogenesis of several granulomatous uveitic entities. B lymphocytes have been identified in the choroidal inflammatory infiltrate in granulomatous uveitis associated with VKH disease [12, 13], with similar findings reported in sympathetic ophthalmia [14–16], multifocal choroiditis [17], and progressive subretinal fibrosis [18]. Additionally, elevated levels of the B-cell chemoattractant CXCL13 have been detected in the aqueous humor of VKH patients [19, 20]. These findings suggest that CXCL13 may serve as an inflammatory biomarker in patients with granulomatous uveitis associated with VKH disease and that B lymphocytes may be pathogenetically important in this uveitis entity.

The predominance of CD20 + B lymphocytes in the uveal inflammatory infiltrate in granulomatous uveitis and the elevated levels of B cell-specific cytokines and chemokines in the aqueous humor in VKH disease provide a strong rationale for targeting B cells in the treatment of these diseases [19–21].

The presence of autoantibodies and T cell receptors against specific autoantigens define autoimmune diseases [6]. Thus, alongside of T lymphocytes, B cells play a central role in the development and propagation of autoimmune diseases, such as rheumatoid arthritis, systemic lupus erythematosus, multiple sclerosis, Sjögren’s syndrome, myasthenia gravis and autoimmune thyroiditis [22–24]. In addition to producing autoantibodies, B cells may capture antigens through their B cell receptor and contribute to autoimmunity by presenting auto- antigens to pathogenic T cells providing T cell help, production of proinflammatory cytokines and formation of ectopic lymphoid structures, a process termed neo-organogenesis [22–24]. Consequently, B cell-targeted therapies have emerged as an effective therapy in patients with autoimmune diseases even in diseases that are believed to be mainly mediated by T cells, such as rheumatoid arthritis and multiple sclerosis [22–24].

Rituximab, a chimeric monoclonal antibody targeting CD20 on B cells, has shown efficacy in treating autoimmune diseases unresponsive to standard treatment [23, 25]. Previous studies have reported rituximab’s effectiveness in various resistant ocular inflammatory conditions [26–34].

This study aims to evaluate the safety and efficacy of rituximab in treating refractory granulomatous uveitis and scleritis and assesses its role in inducing disease quiescence, allowing for reduction in corticosteroid and immunomodulatory medications.

### Patients and methods

The medical records of patients with refractory chronic granulomatous uveitis and scleritis treated with rituximab intravenous infusion at King Abdulaziz University Hospital, Riyadh were retrospectively reviewed from January 2018 to December 2024. This study included the follow-up data of the 9 patients (18 eyes) that were included in our previous publication [33]. Combination conventional immunosuppressive treatment had failed to induce remission in all patients. The study was conducted in adherence with the tenets of the Declaration of Helsinki for research involving human subjects. All patients signed an informed consent for treatment.

Charts were reviewed for demographic data (age and gender), best-corrected Snellen visual acuities (BCVA), applanation tonometry intraocular pressure, results of slit-lamp examination of the anterior segment, results of dilated fundus examination, interval between initial presentation and starting rituximab therapy, ocular complications and duration of follow-up. The outcome variables were visual acuity, control of inflammation, corticosteroid-sparing effect, tapering other immunomodulatory agents and adverse effects.

All patients underwent a protein purified derivative skin test, QuantiFERON tuberculosis testing, and chest radiography. Patients had a general physical examination and laboratory evaluation before each rituximab infusion. Tapering of immunosuppressive therapy was attempted in patients with controlled intraocular inflammation. In general, prednisone therapy was tapered first unless it was medically necessary to first taper a concomitant immunomodulatory therapy owing to drug-related side effects. At each examination, the dose of prescribed concomitant immunosuppressive medication was recorded, and patients were questioned about the development of any adverse effects.

All patients were admitted and rituximab was administered at a dose of 1000 mg per intravenous infusion (375 mg/m² for children) on days 1 and 15, then at 6 months. All patients received 100 mg hydrocortisone intravenously, oral paracetamol, and antihistamines before rituximab infusion to reduce the risk of infusion reactions. Patients with activity following the third dose of rituximab were given additional doses as required. All patients were managed and followed up by one of the authors (AMA).

### Statistical analysis

Snellen visual acuities were converted to the logarithm of the minimum angle of resolution (logMAR) for statistical analysis. The paired sample t-test was used to compare visual acuity before rituximab therapy and at last follow-up. A p-value less than 0.05 indicated statistical significance. Data were analyzed using SPSS version 21.0 (IBM Inc., Chicago, Illinois, USA).

## Results

### Patient demographics

Twenty four patients (47 eyes) were included in this retrospective study. Two patients were male, and 22 were female. The age at presentation ranged from 8 to 68 years (mean 26.2 ± 12.5 years). Tables 1 and 2 presents the detailed demographic and clinical characteristics of the study subjects.

**Table 1:** Demographic and treatment data for chronic recurrent Vogt-Koyanagi-Harada disease (VKH)

Presented in the initial-onset acute uveitic phase with delayed presentation															
#	Diagnosis	Age/Sex	Interval between presentation and Rituximab treatment (months)	Number of Rituximab dose	Interval between 3rd and 4th dose of Rituximab (months	VA before Rituximab treatment	VA 1 year after Rituximab treatment	VA at last visit	Treatment at initial presentation	Daily therapy before Rituximab treatment	Daily therapy after Rituximab treatment	Duration of follow-up after Rituximab treatment (months)			
1	VKH	33/F	28	3		RE 20/60 LE 20/28.5	RE 20/30 LE 20/30	RE 20/30 LE 20/30	3 doses IVMP Mycophenolate mofetil 1000mg bid	Mycophenolate mofetil 1g bid Prednisolone 5mg every other day	Mycophenolate mofetil 500mg bid	24			
2	VKH	30/F	12	3		RE 20/400 LE 20/400	RE 20/100 LE 20/80	RE 20/100 LE 20/40	3 doses IVMP Mycophenolate mofetil 1000mg bid	Mycophenolate mofetil 1 g bid Prednisolone 20 mg	Mycophenolate mofetil 500mg	30			
3	VKH	43/F	9	3		RE 20/28.5 LE 20/40	RE 20/20 LE 20/30	RE 20/25 LE 20/30	3 doses IVMP Mycophenolate mofetil 1000mg bid	Mycophenolate mofetil 1 g bid Prednisolone 20 mg	Mycophenolate mofetil 1g	35			
4	VKH	35/F	12	3		RE CF LE 20/60	RE 20/30 LE 20/20	RE 20/30 LE 20/20	3 doses IVMP Mycophenolate mofetil 1000mg bid	Mycophenolate mofetil 1 g bid Prednisolone 7.5mg	Mycophenolate mofetil 1g	38			
5	VKH	35/F	16	3		RE 20/40 LE 20/40	RE 20/25 LE 20/20	RE 20/25 LE 20/20	3 doses IVMP Mycophenolate mofetil 1000mg bid	Mycophenolate mofetil 1g bid Prednisolone 20 mg	Mycophenolate mofetil 1g bid	18			
6	VKH	8/F	10	4	14	RE 20/100 LE 20/200	RE 20/40 LE 20/60	RE 20/30 LE 20/30	3 doses IVMP Methotrexate 15mg/wk	Methotrexate 15mg/wk Prednisolone 7.5 mg	Methotrexate 5 mg/week	70			
Presented in the chronic recurrent phase															
#	Diagnosis	Age/Sex	Interval between presentation and Rituximab treatment (months)	Number of Rituximab doses	Interval between 3rd and 4th dose of Rituximab (months)	Interval between 3rd and 5th dose of Rituximab (months)	Interval between 3rd and 6th dose of Rituximab (months)	Interval between 3rd and 7th dose of Rituximab (months)	Visual acuity before Rituximab treatment	VA 1 year after Rituximab treatment	VA at last visit	Treatment at initial presentation	Daily therapy before Rituximab treatment	Daily therapy after Rituximab treatment	Duration of follow-up after Rituximab treatment (months)
7	VKH	15/M	3	4	5	20			RE 20/80 LE 20/80	RE 20/80 LE 20/80	RE 20/28.5 LE 20/28.5	Methotrexate 15 mg/wk Prednisolone 60 mg	Mycophenolate mofetil 1500 Prednisolone 30 mg	Mycophenolate mofetil 500 mg Prednisolone 5 mg	30
8	VKH	13/F	39	3					RE 20/200 LE 20/200	RE 20/80 LE 20/30	RE 20/60 LE 20/20	Mycophenolate mofetil 1 g bid Prednisolone 30 mg	Mycophenolate mofetil 500 bid PF bid Prednisolone 5 mg	Mycophenolate mofetil 500 mg	40
9	VKH	20/F	84	3					RE 20/40 LE LP	RE 20/20 LE NLP	RE 20/30 LE NLP	Mycophenolate mofetil 1 g bid Prednisolone 90 mg	Mycophenolate mofetil 1 g bid, PF qid	Off medication	72
10	VKH	28/F	156	7	19	27	38	49	RE 20/40 LE CF	RE 20/28.5 LE CF	RE 20/40 LE CF	Mycophenolate mofetil 1 g bid Prednisolone 60 mg	Mycophenolate mofetil 1g bid Prednisolone 10 mg	Mycophenolate mofetil 1gm bid	53
11	VKH	33/F	96	5	42	54			RE 20/40 LE 20/40	RE 20/30 LE 20/30	RE 20/30 LE 20/20	Mycophenolate mofetil 1 g bid Prednisolone 20 mg	Mycophenolate mofetil 1g bid Prednisolone 7.5 mg PF bid	Mycophenolate mofetil 1500 mg	64
12	VKH	22/F	48	6	7	55	67		RE 20/200 LE CF	RE 20/30 LE CF	RE 20/28.5 LE 20/100	Mycophenolate mofetil 1 g bid Prednisolone 90 mg	Mycophenolate mofetil 1 g bid PF qid	Mycophenolate mofetil 1500 mg	80
13	VKH	18/F	36	4	50				RE 20/400 LE CF	RE 20/100 LE 20/80	RE 20/20 LE 20/80	Mycophenolate mofetil 1 g bid Prednisolone 70 mg	Mycophenolate mofetil 1g bid Prenisolone 10 mg	Mycophenolate mofetil 500 mg bid	75
14	VKH	20/F	60	5	6	28			RE 20/100 LE 20/200	RE 20/20 LE 20/20	RE 20/25 LE 20/20	Mycophenolate mofetil 1 g bid Prednisolone 60 mg	Mycophenolate mofetil 1g bid PF qid	Mycophenolate mofetil 500 mg bid	43
15	VKH	14/F	36	3					RE 20/40 LE 20/40	RE 20/20 LE 20/25	RE 20/25 LE 20/25	Mycophenolate mofetil 1 g bid Prednisolone 45 mg	Mycophenolate mofetil 1g bid PF qid	Mycophenolate mofetil 1 g bid	58
16	VKH	35/F	36	7	15,	23	40	52	RE HM LE 20/200	RE HM LE HM	RE HM LE 20/100	Mycophenolate mofetil 1 g bid Prednisolone 60 mg	Mycophenolate mofetil 1g bid PF qid	Mycophenolate mofetil 1 g bid	50

*VA* visual acuity, *RE* right eye, *LE* left eye, *IVMP* intravenous methylprednisolone, *PF* prednisolone acetate eye drop

**Table 2:** Demographic and treatment data for other diagnoses

#	Diagnosis	Age/Sex	Interval between presentation and Rituximab treatment (months)	Number of Rituximab doses	Interval between 3rd and 4th dose of Rituximab (months)	Visual acuity before Rituximab treatment	VA 1 year after Rituximab treatment	Visual acuity at last visit	Treatment at initial presentation	Daily therapy before Rituximab treatment	Daily therapy after Rituximab treatment	Duration of follow-up after Rituximab treatment (months)
17	Scleritis	29/F	4	3		RE 20/20 LE 20/25	RE 20/20 LE 20/20	RE 20/20 LE 20/20	Azathioprine 125 mg Prednisolone 20 mg	Azathioprine 125 mg Prednisolone 5 mg	Azathioprine 100 mg	16
18	Scleritis	68/F	3	3		RE 20/25 LE 20/40	RE 20/20 LE 20/30	RE 20/20 LE 20/30	Topical PF qid, Voltaren qid	Mycophenolate mofetil 1g bid Prednisolone 20 mg Volatren qid	Mycophenolate mofetil 1500 mg	12
19	Granulamtous Panuveitis	19/F	120	3		RE CF LE CF	RE 20/30 LE CF	RE 20/25 LE 20/400	Mycophenolate mofetil 1 g bid Prednisolone 70 mg	Prednisolone 60 mg mycophenolate mofetil 1 g bid PF qid	Prednisolone 5 mg	25
20	Granulamtous Panuveitis	16/F	14	3		RE 20/25 LE 20/20	RE 20/20 LE 20/20	RE 20/20 LE 20/70	Mycophenolate mofetil 1 g bid Prednisolone 60 mg	Mycophenolate mofetil 1g bid Prednisolone 10 mg	Off medications	41
21	Granulamtous Panuveitis	14/F	8	3		RE 20/20 LE 20/20	RE 20/20 LE 20/25	RE 20/20 LE 20/20	IVMP Mycophenolate mofetil 1 g bid	Mycophenolate mofetil 1500 mg Prednisolone 7.5 mg	Mycophenolate mofetil 500 mg	24
22	MS related granulomatous uveitis	26/F	41	3		RE 20/25 LE 20/20	RE 20/20 LE 20/20	RE 20/20 LE 20/20	Mycophenolate mofetil 1 g bid Prednisolone 60 mg	Mycophenolate mofetil 1g bid Prednisolone 7.5 mg PF qid	Mycophenolate mofetil 500 mg bid	19
23	MS related granulomatous uveitis	28 F	50	4	6	RE 20/80 LE 20/400	RE 20/80 LE CF	RE 20/30 LE 20/80	IVMP Mycophenolate mofetil 1 g bid	Mycophenolate mofetil 1 g Prednisolone 20 mg	Mycophenolate mofetil 1 g bid Pred 5 mg	18
24	Progressive Subretinal Fibrosis	27/M	36	3		RE 20/200 LE 20/200	RE 20/100 LE 20/60	RE 20/40 LE 20/60	Mycophenolate mofetil 1 g bid Prednisolone 80 mg	Mycophenolate mofetil 1500 mg Prednisolone 5 mg	Mycophenolate mofetil 500 mg	45

*VA *visual acuity, *RE* right eye, *LE *left eye, *MS* multiple sclerosis, *IVMP* intravenous methylprednisolone, *PF* prednisolone acetate eye drop

The diagnosis of these patients included chronic recurrent uveitis associated with VKH disease (16 cases, 66.6%), idiopathic granulomatous uveitis (3 cases, 12.5%), granulomatosis with polyangiitis (GPA) associated scleritis (2 cases, 8.3%), multiple sclerosis (MS)-related uveitis (2 cases, 8.3%), and bilateral progressive subretinal fibrosis (1 case, 4.16%). Mean visual acuity at presentation was 0.93 ± 0.67 logMAR (Snellen: 20/160).

Of 16 patients (32 eyes) with chronic recurrent VKH disease that were included, six patients (12 eyes) presented as initial-onset delayed VKH (i.e.; had anterior segment inflammation) (9) and later progressed to chronic recurrent disease. The remaining ten patients (20 eyes) presented with chronic recurrent granulomatous uveitis and had “sunset glow” fundus at presentation [32]. Baseline visual acuity for all VKH patients was 0.88 ± 0.65 logMAR (Snellen equivalent 20/150). Granulomatous anterior uveitis with mutton-fat keratic precipitates, anterior chamber reaction (≥ 2 + cells) and “sunset glow fundus” were noted in all 32 eyes.

Baseline visual acuity in the other eight patients (15 eyes) was 0.50 ± 0.63 logMAR (Snellen 20/60). Examination findings in the 3 patients with idiopathic granulomatous uveitis included mutton-fat keratic precipitates and anterior chamber reaction (≥ 2 + cells) along with posterior synechiae in 6 eyes, optic nerve head (ONH) hyperemia in 6 eyes, vitritis in 4 eyes, and snowballs in two eyes. In the two patients with MS-related uveitis, mutton-fat keratic precipitates and anterior chamber reaction (≥ 2 + cells) along with posterior synechiae were present in all four eyes, vitritis in two eyes, ONH hyperemia in two eyes, and peripheral vascular sheathing in all four eyes. In the two patients with GPA-associated scleritis, diffuse scleritis was observed in two eyes of one patient, while the other had nodular sectoral scleritis in one eye. In the patient with bilateral progressive subretinal fibrosis findings included choroidal lesions and scarring in both eyes along with subretinal fibrosis, and macular neovascular membrane (MNV) in one eye.

Similar to the VKH cohort, all patients failed conventional therapy, leading to progressive disease and complications. One patient with idiopathic granulomatous uveitis (patient 19) developed cystoid macular edema in both eyes, requiring multiple dexamethasone injections. Another patient with diffuse progressive subretinal fibrosis (patient 24) developed severe MNV requiring Iluvien injection.

### Initial therapy

Of 16 patients with VKH disease, the 6 patients presenting as initial-onset acute VKH disease with delayed presentation received intravenous methylprednisolone (1 g/day for adults, 15–30 mg/kg for children) along with mycophenolate mofetil (1 g bid) or methotrexate (15 mg/week for children) as primary treatment. The other ten patients with chronic recurrent VKH disease at presentation were treated with oral corticosteroids as initial therapy, in combination with mycophenolate mofetil (1 g bid in adults) or methotrexate (15 mg/week in children). Three patients with granulomatous panuveitis received mycophenolate mofetil (1 g bid), with one patient receiving intravenous methylprednisolone and two receiving oral corticosteroids. Similarly, two patients with MS-related uveitis were treated, with one receiving intravenous methylprednisolone and mycophenolate mofetil, while the other received oral corticosteroids with mycophenolate mofetil. One patient with progressive subretinal fibrosis was treated with oral corticosteroids as initial therapy in addition to mycophenolate mofetil (1 g bid). Additionally, one of the two patients with scleritis received azathioprine as primary treatment in conjunction with oral steroids.

Despite treatment with systemic corticosteroids in combination with nonsteroidal immunomodulatory therapy, all patients experienced multiple flare-ups requiring medication escalation. However, due to persistent disease activity despite conventional treatment, all patients were started on rituximab therapy according to the protocol described earlier.

### Rituximab therapy outcomes

The interval between initial presentation and initiation of rituximab therapy ranged from 3 to 156 months (mean 39.8 ± 39.2 months). Fifteen patients (62.5%) were controlled after 3 doses of rituximab, while 9 patients (37.5%) required additional doses: two patients required only one additional 4th dose, three patients required a further 5th dose, while two patients required 6 total doses, and two patients required 7 total doses. Tables 1 and 2 show the intervals between rituximab doses in VKH patients and in other inflammatory entities. Visual acuity at 1 year after rituximab significantly improved from 0.93 ± 0.67 logMAR (Snellen: 20/160) to 0.63 ± 0.83 logMAR (Snellen: 20/80) (*p* < 0.001). Follow-up ranged from 12 to 80 months (mean 41 ± 20.5). At the last follow-up, best-corrected visual acuity (BCVA) improved to 0.46 ± 0.73 logMAR (Snellen: 20/60) (*p* < 0.001). Furthermore, rituximab was successful in reducing corticosteroid requirement, enabling complete oral steroid discontinuation in 21 patients (87.5%), with the remaining 3 patients requiring ≤ 7.5 mg per day. Mycophenolate mofetil dosage significantly decreased from a mean of 1840 ± 323 mg to 1045 ± 688 mg daily (*p* < 0.001). Two patients were able to completely discontinue medications. (Tables 1 and 2). All patients were controlled with no flare ups on their last follow-up. Five eyes underwent uneventful cataract surgery post-rituximab treatment. There was no rituximab-related complications during follow-up.

Among patients with refractory chronic recurrent VKH disease, those who presented to our institute in the acute uveitic phase with delayed presentation (i.e.; anterior segment inflammation) (*n* = 6) had better visual outcome than those who presented in the chronic recurrent phase (*n* = 10) with mean final BCVA 0.19 ± 0.17 logMAR (Snellen 20/30) and l0.54 ± 0.83 logMAR (Snellen 20/70), respectively (*p* = 0.177).

At the final visit, patients in the acute uveitic phase with delayed presentation (i.e., anterior segment inflammation) (*n* = 6) had a better final mean BCVA of 0.19 ± 0.17 logMAR (Snellen 20/30) compared to those who presented in the chronic recurrent phase (*n* = 10) who had a final mean BCVA of 0.54 ± 0.83 logMAR (Snellen 20/70), However, the difference was not significant (*p* = 0.08).

## Discussion

In agreement with previous studies [26, 27, 33–36], this study highlights the potential of rituximab as an effective and safe therapeutic option for patients suffering from refractory granulomatous uveitis and scleritis. The study’s results contribute valuable insights into the expanding role of B-cell-targeted therapies in managing ocular inflammatory diseases that are resistant to conventional treatments, and is in agreement with other studies that have shown efficacy of rituximab treatment in refractory uveitis and scleritis juvenile [26–34].

Several studies have highlighted the potential role of B lymphocytes in the pathogenesis of granulomatous uveitis associated with VKH disease [12, 13]. B lymphocytes have been identified in the choroidal inflammatory infiltrate in other granulomatous uveitic conditions such as sympathetic ophthalmia [14–16], multifocal choroiditis [17], and progressive subretinal fibrosis [18]. Additionally, elevated levels of the B cell chemoattractant CXCL13 and the cytokines a pro-inflammatory inducing ligand (APRIL) and the B-cell-activating factor of the TNF family (BAFF) have been detected in the aqueous humor of VKH patients [19, 21]. These cytokines and chemokines are known to promote B cell survival, differentiation, and antibody production, suggesting a critical role for B cells in the local immune response and disease progression in VKH [19–21].

The predominance of CD20 + B lymphocytes in the uveal inflammatory infiltrate and the elevated levels of B cell-specific cytokines and chemokines in VKH patients provide a strong rationale for targeting B cells in the treatment of this disease. Our clinical data support the hypothesis that B cell depletion with rituximab is a rational and effective approach for managing refractory chronic recurrent uveitis associated with VKH disease and other granulomatous uveitis entities. The significant improvement in visual acuity and the stabilization of disease activity observed in our study further reinforce the potential of rituximab as a valuable therapeutic option for patients with difficult-to-treat granulomatous uveitis and scleritis.

Among patients who presented with the initial-onset acute stage of VKH disease with delayed presentation then progressed to chronic recurrent disease, rituximab significantly improved visual outcomes, with visual acuity improving from a baseline of 20/100 to 20/30 at the final follow-up. These patients had better outcomes compared to patients presenting in the chronic recurrent phase. The transition of these patients to a chronic recurrent stage despite aggressive initial treatment is most likely because these patients presented outside the therapeutic window of opportunity [9]. For patients initially presenting with chronic recurrent VKH, rituximab also offered substantial benefits although the initial visual improvement was modest, with a baseline visual acuity of 20/200 improving to 20/90 one year after treatment. Continued follow-up showed further improvement to 20/70. The need for additional rituximab doses in seven out of ten of these patients indicates that while rituximab is effective, its administration may need to be tailored to individual patient responses, potentially requiring repeated dosing to maintain disease control. The ability to reduce the burden of corticosteroid and immunosuppressive therapy in these patients further supports the utility of rituximab as part of a long-term management strategy for chronic VKH disease.

The study also sheds light on rituximab’s effectiveness in treating other forms of granulomatous uveitis, including idiopathic granulomatous uveitis, multiple sclerosis-related uveitis, progressive subretinal fibrosis, and scleritis. Achievement of disease quiescence and consistent visual improvement across these varied conditions suggests that rituximab’s mechanism of action, which targets B cells, is effective in a broad spectrum of uveitic diseases. This is particularly relevant as B cells are increasingly recognized as key players in the pathogenesis of autoimmune diseases traditionally thought to be mediated primarily by T cells [6, 22–24].

Safety is a critical consideration when evaluating any long-term treatment, particularly for conditions like uveitis that require prolonged therapy to prevent relapses. Although infusion reactions, macular edema, hematological events, interstitial lung fibrosis, infectious complications, and an increased risk of malignancy have been reported in previous studies [36], rituximab was well-tolerated in this cohort with no significant drug-related adverse events reported throughout follow-up. The pre-treatment regimen of hydrocortisone, paracetamol, and antihistamines appears to have effectively minimized the risk of infusion reactions, which are a common concern with rituximab therapy. This favorable safety profile is consistent with other studies of rituximab in autoimmune diseases, where it has been shown to be safe even with long-term use [37].

While the results of this study are promising, several limitations must be acknowledged. The retrospective nature of the study and the relatively small sample size limit the generalizability of the findings. Moreover, the study population was heterogeneous, including various uveitic entities with different underlying pathologies, which might have influenced the outcomes. Additionally, the long and varied intervals between initial presentation and rituximab initiation could introduce bias, as the natural disease course and other treatments might have impacted the results. Future research should focus on prospective, randomized controlled trials with larger, more homogeneous cohorts to better evaluate the efficacy and safety of rituximab in granulomatous uveitis and ocular inflammation. These studies should also explore optimal dosing regimens, the potential need for maintenance therapy, and the long-term outcomes associated with rituximab treatment. Moreover, understanding the biomarkers that predict response to Rituximab could help personalize therapy and identify patients who are most likely to benefit from this treatment.

##  Conclusion

In conclusion, this study provides valuable evidence supporting the use of rituximab as a safe and effective treatment for refractory granulomatous uveitis and scleritis. The significant improvements in visual acuity and the ability to achieve disease quiescence and reduce the need for other immunosuppressive therapies make rituximab a promising option for patients with resistant granulomatous ocular inflammation.

## Data Availability

No datasets were generated or analysed during the current study.

## Abbreviations

VKH: Voght-Koyanagi-Harada
MS: Multiple sclerosis
GPA: Granulomatosis with polyangiitis
MNV: Macular neovascular
BCVA: Best corrected visual acuity
ONH: Optic nerve head
